# Plastic Fillings

**Published:** 1877-09

**Authors:** C. L. Anderson

**Affiliations:** Springfield, Mass.


### ARTICLE IV.

### *Plastic Fillings.*

Read before the Connecticut Valley Dental Association, June 13, by Dr. C. L. Anderson, of Springfield, Mass.

The remarks about to be offered on this subject are not intended to be exhaustive in their discussion of the theme, but merely to present some of the more salient points which are engaging the minds of men eminent in the profession, and which may serve as a nucleus for profitable discussion and thought in unoccupied hours.

Jewelry to adorn the human form divine is now at this advanced stage of civilization considered a necessity, but the idea seems to be growing on the abler men of our profession that its display in the mouths of our patients is not so imperatively demanded; and that notwithstanding the strides that dental science and art have made, we as yet lack that without which we cannot claim for ourselves any reasonable proximity to success, as far as our means for accomplishing the chief ends of dentistry are concerned—that of the preservation of the natural teeth intact, or nearly so—which calls for a material that shall subserve the office of a preserver, and not at the same time direct attention to the fact that tooth structure is lost, and has been replaced by an artificial substitute.

Therefore it is to be regretted that the material most frequently made use of for this purpose is on many accounts a most objectionable one. Besides its appearance, gold is undesirable from the fact of the extreme danger of insertion in many teeth on account of their frailty, and in others of a peculiar construction, to the preservation of which gold seems inimical. Besides, there is a large class of patients who cannot bear the suffering and tedium attendant upon the insertion of gold plugs; the more refined the practice, the larger in proportion will this class of patients be, and consequently the higher the skill demanded of the operator. The higher the mental development, the more susceptible to nervous influence is the organism.

Electro-galvanic action between tooth structure and filling materials, is at present a mooted theme as regards the extent of the action and the degree of injury resulting; but that there is such a thing I think no scientific dentist will deny, for to many minds there are phenomena which are unexplainable except with this hypothesis, and others which point directly to this force as a disturbing element to success. Experiments made with a view of determining the *truth*, establish beyond the shadow of a doubt the *fact*, and the future will probably reveal the extent of its operations.

As the result of these experiments, gold and dentine present the strongest intensity of action, accompanied with the greatest deviation of the galvanometer.

As the result of methodically kept records, extending five, ten, fifteen, and even twenty and thirty years back, among other valuable facts, one in regard to gold has been adduced —that there are many teeth in which gold has been inserted in the most skilled manner, not once but two or three times, and these plugs have as often failed without the slightest indication of decay; simply becoming loose and dropping out, or loosened by brushing or some equally harmless cause. In other teeth plugs fail which have the greatest care bestowed on their insertion and after treatment, and no reason, to many minds, seems assignable. The lateral incisor is a striking example of this fact. As these and other facts are becoming more and more evident, the conscientious practitioner begins to anxiously peer into the pregnant future, to glean, if he may, something which shall lead him to the goal where lies a material for plugging teeth, which shall possess, not only the requisite impermeability, indestructibility, adaptability; resistance to mechanical, medicinal and chemical action, but also the proper color, and whose working qualities shall not demand for a perfect plug the tedious and often painful operations that gold does.

Along with the conviction that is so rapidly gaining ground that soft, non-cohesive gold, is, on many accounts, superior to the cohesive form, comes the candid confession that we need an easily manipulated permanent natural appearing material for the better practice of our profession, and the furtherance of its truest interest.

It must be evident that the necessity exists, and as "necessity is the mother of invention," we may hope and expect that some important advance will be made, even if the goal is not reached in our day.

A few words as to the materials most used in a plastic form, and the modes of their manipulation:

Amalgam, our professional Hydra, is probably the most used and misused material we have; but in its best forms it fills a place no other can, and without it many crownless roots would have been extracted, instead of doing valuable service in mouths, where, although perhaps hastily placed, it has outlasted many elegant gold fillings.

From experiments it has been found that the proper mode is to weigh our amalgams, having the mercury in one pan of the scales a little in excess of the ground mixture in the other. We determine the amount of mercury by the size of the cavity or cavities to be filled, taking enough to about fill one-half the required space. After weighing comes the mixing, and a small mortar (Fletcher's is excellent) is the most convenient and rapid mode; besides the discoloration of the skin, and a suspicion of uncleanliness suggested to the patient by mixing in the palm, there is a possible danger of some constitutional effects from its prolonged practice.

Have the cavity perfectly dry, a very important condition of success. Hold the amalgam in one hand, working with the other, thus keeping it at a proper temperature (same as the tooth,) preserving its working qualities much longer. Always *tap*, never *rub* in, plastic fillings of any kind, so that the plug shall be uniform. When the cavity is all but filled, take the surplus amalgam and squeeze out the mercury, so as to make it very dry, and use this "bottom" to end off with, tapping in thoroughly to draw out the mercury; then finish off, for which purpose a pair of pliers and small pieces of spunk are admirable. The mercury may be squeezed out between the fingers, or in a chamois with a pair of smooth, flat-nozed pliers. Washing, and *preliminary* squeezing are detrimental, and the proportions given indicate the proper mixture to obtain the best working qualities. In extensive building, a little more plasticity is not objectionable. When sufficiently hard all amalgam fillings should be finished off the same as gold, with pumice, etc.

The use of amalgams is of course limited to the molars and bicuspids; for the incisors two materials are admissible, —gutta-percha and the oxy-chloride of zinc. Of the two gutta-percha is by far the most desirable, and its merits as a perserver of bad teeth are not nearly so well appreciated as they should be.

Take any tooth, decayed on any surface except the masticating where a good gold plug has failed, and try gutta-percha and await the result. It will often outlast the gold and if it shows signs of failure, it is easily removed and replaced, without any extensive excavations and with very little of the trouble and expense attendant upon the re-insertion of gold. It is nearer the color of the tooth, and often matches so perfectly that its presence is not detected. The fact that gutta-percha is in many instances a permanent plug has been proved. The poorer in structure the teeth, more satisfactory the result, when compared with that obtained by the use of gold. The best form at present is Johnston's "Premium Stopping" (N. Y.;) it approaches nearer to the old forms of gutta-percha which proved so reliable. When used it should be cut in small pieces and warmed over wet, not dry heat. Dry heat distroys its toughness and working qualities. It should be heated until in a semi-pasty condition, and inserted with warm, not hot instruments, and very neatly finished down with no overlapped edges to "ruff" up and present lodgments for food and invite decay.

At the present time oxy-chlorides are receiving considerable attention and vigorous efforts are instituted to bring them in a more durable form. Like amalgam their names are out legion. Fletcher's seems to be peculiarly adapted to capping pulps,* and in this connection the practice of filling root canals with this material should be condemned as likely to cause alveolar necrosis by sleeping out, especially in lower teeth. Poulson's German Cement is much better

*Dr. A. has evidently confounded Fletcher's White Enamel with Fletcher's Artificial Dentine. The former is an oxy-chloride and could scarcely be used for "capping pulps;" while the latter contains no chloride, and consequently is perfectly free from irritant action.—Editor. —*Dental Advertiser.*

than Cement Plombe for general use. If one fact could be definitely determined, the usefulness of this material would be much enhanced: In what mouths does oxy-chloride of zinc stand the best? As long as this is not known we must write " unreliable " as our verdict. When it does stand, it is *ne plus ultra*; but when it fails it melts like ice.

As an outgrowth of the movement to bring it out in a more enduring form, comes the "talcking method," so called. The principle of this is surface hardness produced by the use of French chalk in a heated state, and rubbed for a length of time over the face of the filling. Fillings inserted in this manner give promise of good results, but more time is necessary to test it thoroughly. It starts off well, and we may cherish the hope that it is a step in the advance for which we are looking.

And now that the necessity of some durable plastic filling material is acknowledged, and that we have already material which properly used can be made of great service, let us make a fair trial of them and so help towards the grand result! We do not want to rush blindly into this matter, and by our zeal destroy the force of our efforts, but *judgment* is demanded in this as in all departments of the " healing art."— *Dental Advertiser*.

